# The immune cell infiltration-associated molecular subtypes and gene signature predict prognosis for osteosarcoma patients

**DOI:** 10.1038/s41598-024-55890-0

**Published:** 2024-03-02

**Authors:** Bin Liu, Xiang-Yang Liu, Guo-Ping Wang, Yi-Xin Chen

**Affiliations:** 1 Department of Spine Surgery, Hunan Provincial People’s Hospital (The First-Affiliated Hospital of Hunan Normal University), Changsha, 410005 Hunan China; 2 Department of Rehabilitation Medicine, Xiangya Hospital of Central South University, No. 87, Xiangya Road, Changsha, 410008 Hunan China

**Keywords:** Cancer, Cell biology, Immunology, Oncology

## Abstract

Host immune dysregulation involves in the initiation and development of osteosarcoma (OS). However, the exact role of immune cells in OS remains unknown. We aimed to distinguish the molecular subtypes and establish a prognostic model in OS patients based on immunocyte infiltration. The gene expression profile and corresponding clinical feature of OS patients were obtained from TARGET and GSE21257 datasets. MCP-counter and univariate Cox regression analyses were applied to identify immune cell infiltration-related molecular subgroups. Functional enrichment analysis and immunocyte infiltration analysis were performed between two subgroups. Furthermore, Cox regression and LASSO analyses were performed to establish the prognostic model for the prediction of prognosis and metastasis in OS patients. The subgroup with low infiltration of monocytic lineage (ML) was related to bad prognosis in OS patients. 435 DEGs were screened between the two subgroups. Functional enrichment analysis revealed these DEGs were involved in immune- and inflammation-related pathways. Three important genes (including TERT, CCDC26, and IL2RA) were identified to establish the prognostic model. The risk model had good prognostic performance for the prediction of metastasis and overall survival in OS patients. A novel stratification system was established based on ML-related signature. The risk model could predict the metastasis and prognosis in OS patients. Our findings offered a novel sight for the prognosis and development of OS.

## Introduction

Human osteosarcoma (OS) is an aggressive malignant tumor of bone that occurs mainly in adolescents and young adults^1^. Recent research shows that 60% of OS patients are young, and it is the leading cause of death in this group^2^. OS occurs primarily around the growth plates of bones^3^. The majority of OS patients have metastases to the lungs. Although the progress of clinical therapy in recent years has greatly benefited OS patients, and the 5-year survival rate of patients has significantly improved. However, patients with recurrent or metastatic disease often have a poor prognosis^4–6^. In addition, less than 20% of patients with OS treated with surgery alone survived^7^. Therefore, identifying novel markers that can predict treatment sensitivity and clinical outcomes in OS patients is imperative to effectively improve the survival rate of OS patients.

Immune cell infiltration involves in the bone homeostasis^8^. In addition to skeletal stromal cells, the complex skeletal microenvironment, including bone marrow immune cells, can promote or prevent the progression of bone disease^9^. Host immune dysregulation is related to initiation and progression of OS. T lymphocytes, monocytes, and macrophages are the main subsets of the immune microenvironment of OS^10,11^. Osteosarcoma cells establish an immune microenvironment conducive to tumor metastasis, drug resistance, and growth by controlling the differentiation and recruitment of immune-infiltrating cells^12^. In addition, T cell exhaustion contributes to the development and progression of OS^13^. Immunotherapy is a promising treatment for human malignancies that can improve our understanding of the immune response in OS patients. In recent years, the development of immune-related biomarkers has contributed to a significant increase in the number of patients benefiting from immunotherapy^14^. Some researchers have identified immune-associated biomarkers and prognostic signature for OS patients^15,16^. Low immune score is closely related to poor prognosis in OS patients^17^. Therefore, it is crucial to systematically assess the immunocyte infiltration and determine the function and distribution of immune cells to improve the efficacy of immunotherapy in patients with OS.

We aimed to screen the potential immune cell infiltration-related genes as markers related to risk stratification in OS. We performed the univariate Cox regression analysis to screen prognosis-associated immune cell. Differentially expressed genes (DEGs) were analyzed between the immune cell infiltration-related molecular subtypes. Then, a series of bioinformatics analysis methods, including enrichment analysis and immunocyte infiltration were performed to explore the potential molecular mechanisms involved. Finally, a risk model was established based on immunocyte infiltration-associated genes for the prediction OS prognosis.

## Methods

### Data collection and preprocessing

We downloaded the transcriptome data and corresponding clinical information of 88 OS samples from the TARGET database (https://ocg.cancer.gov/programs/target) as the discover cohort. The transcriptome data of GSE21257 dataset and clinical data of 53 OS samples were collected from the Gene Expression Omnibus (GEO) database (https://www.ncbi.nlm.nih.gov/geo/), and as the validation cohort. Prior to the data analysis, the probe name was converted into the corresponding gene symbols and performed data batch normalization.

### Immunocyte infiltration

Microenvironment Cell Populations-counter (MCP-counter) is a robust algorithm, which accomplishes the quantification of the proportion of stromal and immune cells in heterogeneous tissues based on the gene expression profiling^18^. The level of immunocyte infiltration in OS samples was quantified through the MCP-counter. In addition, the ESTIMATE score, immune score, stromal score and Tumor purity were analyzed through ESTIMATE algorithm^19^. We used the Kaplan–Meier survival analysis to estimate the association between overall survival and immune cell infiltration level of OS patients. A *p* value < 0.05 indicates the significant difference.

### DEGs and enrichment analysis

First, we calculated the monocytic lineage score for each OS patient using the MCP-counter algorithm. We divided OS patients into low-monocytic lineage (LML) and high-monocytic lineage (HML) subgroups based on the median value of monocytic lineage score. The “limma” package of R was used to screen DEGs between LML and HML subgroups. The adjusted *p* < 0.05 and ∣logFC∣ ≥ 1 as the cutoff values to identify the DEGs^20^. The DEGs results were visualized by using the “heatmap” and “ggplot2” packages. The “clusterProfiler” package of R was used to carry out the Gene ontology (GO) and Kyoto Encyclopedia of Genes and Genomes (KEGG) enrichment analyses of DEGs, the results were visualized by using the “ggplot2” package of R, term with a *p* < 0.05 was indicated statistical difference.

### The prognostic model was constructed based on monocytic lineage-related genes

First, we carried out the univariate Cox analysis to identify the survival-associated genes based on the above DEGs, and *p* < 0.05 as the cutoff value. The least absolute shrinkage and selection operator (LASSO) Cox regression analysis was carried out by using the “survival” and “glmnet” packages. The multivariate Cox regression analysis was performed to screen independent risk factors related to prognosis. The risk score for each patients was calculated based on the following formula: risk score = coefficient of Gene A × expression of gene A + coefficient of Gene N × expression of gene N^21^. The performance of the prognostic model was assessed through receiver operating characteristic curve and the Kaplan–Meier survival analysis. Based on the results of COX regression analysis, a nomogram was constructed to predict overall survival in OS patients. Nomogram and calibration curve were generated using the “ggplot2” and “survival R” packages.

### GeneMANIA database

GeneMANIA (http://genemania.org) is a versatile and intuitive online platform that facilitates the generation of hypotheses pertaining to gene functionality^22^. To evaluate the potential functions of signature genes, GENEMANIA was utilized to construct a gene interaction network.

### Tumor immune single cell hub (TISCH)

The TISCH database (http://tisch.comp-genomics.org/gallery/) is an extensive compilation of single-cell RNA sequencing data. It offers valuable insights into the diverse nature of the tumor microenvironment. Leveraging this database, we embarked on an exploration of the heterogeneity within the tumor microenvironment across a range of cell types.

### Statistical analysis

Univariate and multivariate Cox regression analyses were applied to screen prognostic biomarkers. The comparison between two groups was achieved by Mann–Whitney test. The overall survival between the two groups was analyzed by using Kaplan–Meier analysis. The statistical analysis was performed by using R software (v3.5.3), a *p* < 0.05 represented statistical difference.

## Results

### ML was related to the prognosis of OS patients

**Figure 1:**
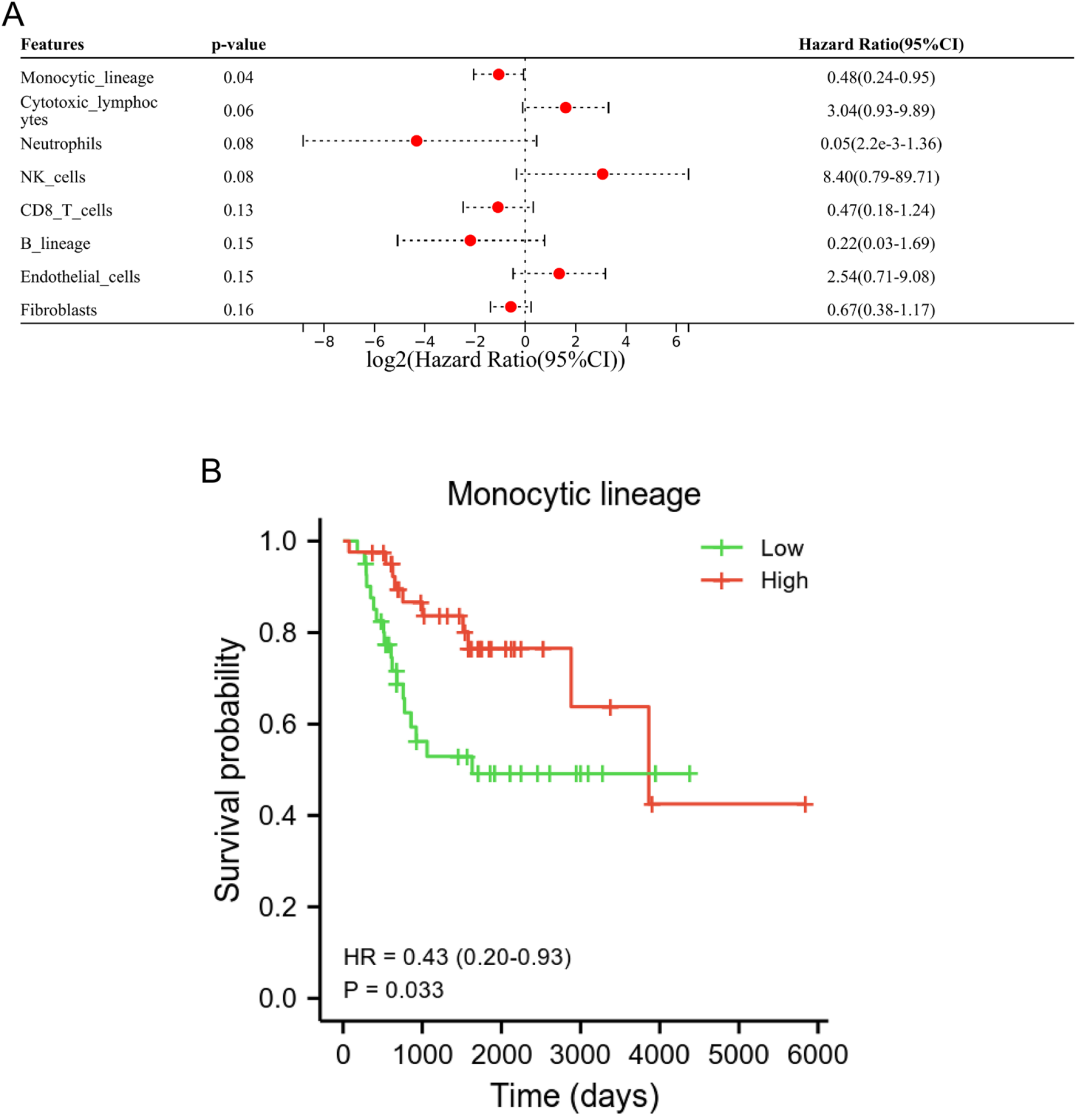
The ML was related to a bad prognosis in patients with OS. (**A**) The univariate Cox analysis of six immune cells and two stromal cells based on TARGET database. (**B**) The Kaplan–Meier analysis showed that the level of ML was significantly associated with prognosis in patients.

In our study, the MCP-counter algorithm and univariate and univariate Cox regression analysis were performed to identify the survival-associated immune cells. The results of univariate Cox regression analysis showed that ML was a survival-related immune cell for OS patients (Fig. 1A). In addition, Kaplan–Meier analysis indicated that low level of ML was related to a poorer prognosis for OS patients (p = 0.033, Fig. 1B).

### Analysis of DEGs and potential signaling pathways between two subgroups

**Figure 2:**
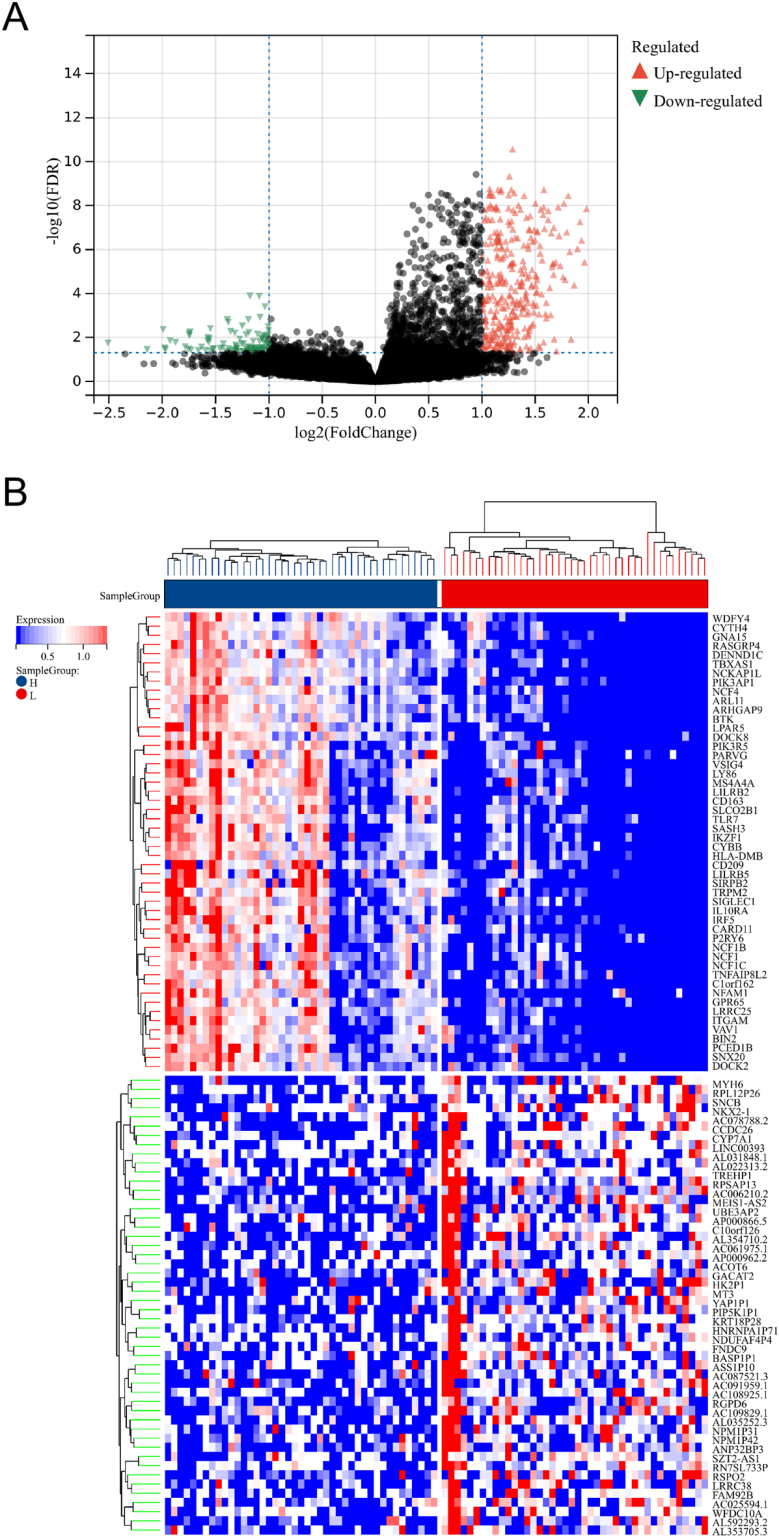
Identification of DEGs between the LML and HML subgroups. (**A**) The volcano plot depicted the DEGs between the LML and HML subgroups. The green dots represented down-regulated genes, whereas the red dots represented up-regulated genes. (**B**) Heatmap plot depicted the top 50 DEGs between the LML and HML subgroups.

**Figure 3:**
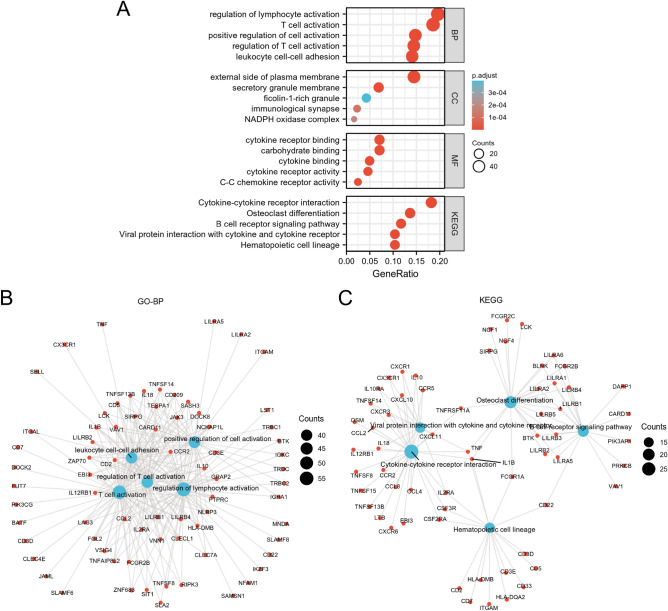
Enrichment analysis of DEGs. (**A**) Bubble plots depicted the results of GO and KEGG. The network diagram depicted the immune- and inflammation-related GO-BP terms (**B**) and KEGG pathways (**C**). The blue nodes represented GO-BP terms or KEGG pathways, red nodes represented genes involved in the pathways.

A total of 435 DEGs were identified following DEGs analysis (Fig. 2A,B). Compared with the LML, 101 genes were downregulated and 334 genes were upregulated in the HML group. In the term of biological processes (BP), these DEGs were involved in regulation of lymphocyte activation, T cell activation, positive regulation of cell activation, regulation of T cell activation, leukocyte cell–cell adhesion, etc. In the term of cellular components (CC), DEGs were involved in external side of plasma membrane, secretory granule membrane, ficolin-1-rich granule, immunological synapse, NADPH oxidase complex, etc. In the term of molecular functions (MF), DEGs were significantly enriched in carbohydrate binding, cytokine receptor binding, cytokine binding, cytokine receptor activity, C–C chemokine receptor activity, etc. In the term of KEGG, DEGs were mainly enriched in hematopoietic cell lineage, osteoclast differentiation, cytokine-cytokine receptor interaction, B cell receptor signaling pathway, etc. (Fig. 3).

### Immunocyte infiltration

**Figure 4:**
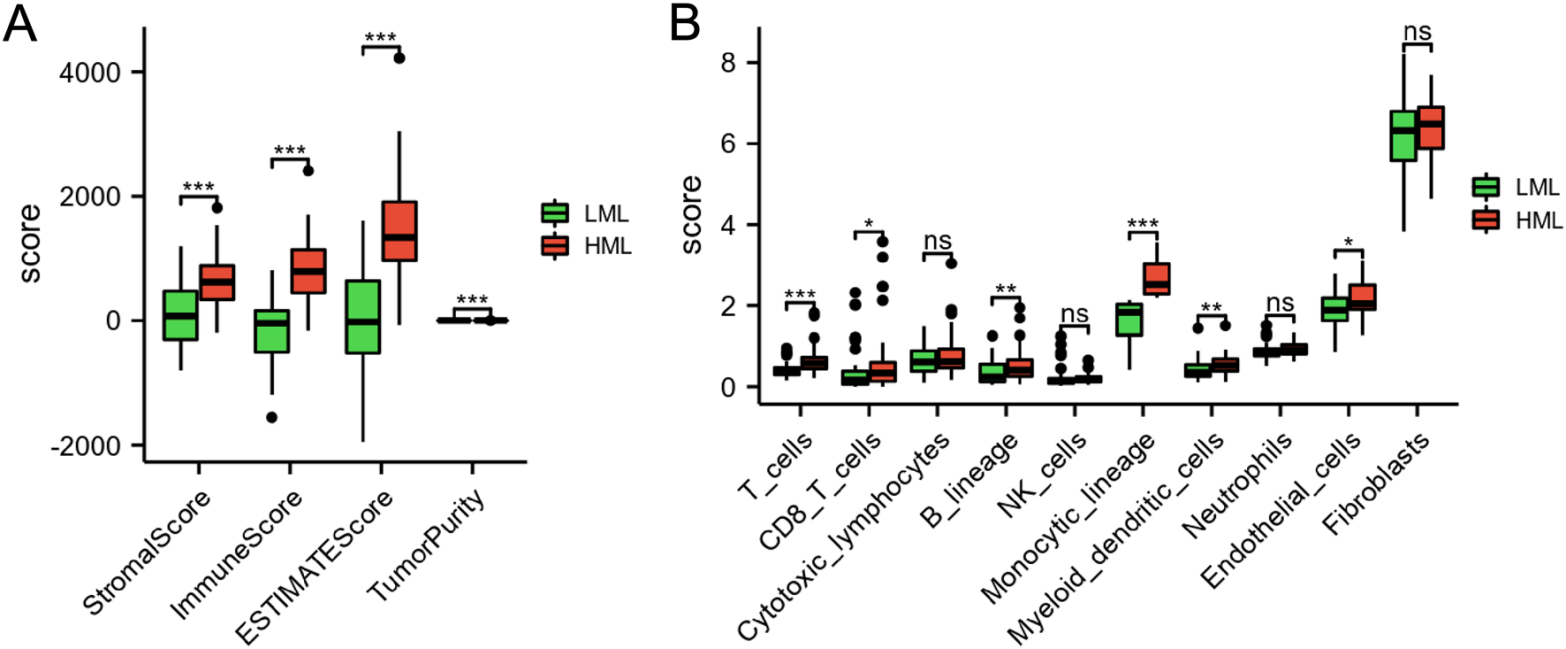
The landscape of immunocyte infiltration levels in the two subgroups. (**A**) The comparisons of stromal score, immune score, ESTIMATE score, and tumor purity between the LML and HML subgroups. (**B**) The comparisons of immunocyte infiltration levels between the LML and HML subgroups. *p < 0.05, **p < 0.01, and ***p < 0.001.

We analyzed the immune status between the LML and HML subgroups to decipher the immune microenvironment in OS. As shown in Fig. 4A, compared to the HML group, the stromal score, immune score, and ESTIMATE score were decreased in the LML group (*p* < 0.01), while tumor purity was significantly increased in the LML group (*p* < 0.01). Compared with the HML group, the endothelial cells, myeloid dendritic cells, monocytic lineage, B lineage, T cells, and CD8 T cells levels were significantly decreased in the LML group (*p* < 0.05) (Fig. 4B).

### Construction and assessment of the prognostic risk model

**Figure 5:**
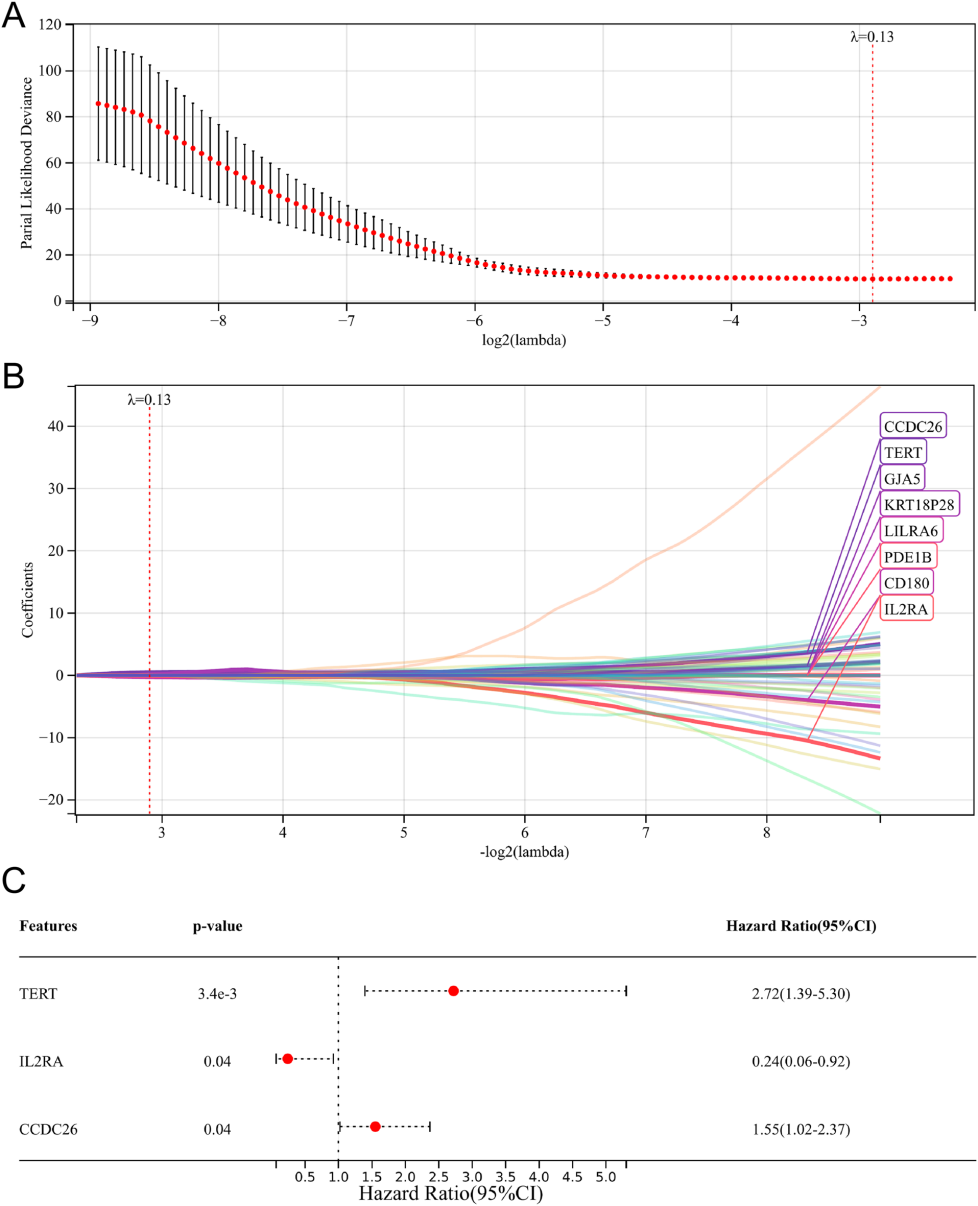
Establishment of ML-associated prognostic model. (**A**, **B**) LASSO regression analysis. (**C**) Multivariate Cox regression analysis.

Following the univariate Cox regression analysis, we screened 122 overall survival-related genes (Table S1). Subsequently, the LASSO Cox regression analysis identified eight genes (CCDC26, TERT, GJA5, KRT18P28, LILRA6, PDE1B, CD180, and IL2RA) for the multivariate Cox regression analysis (Fig. 5A,B). Finally, three important overall survival-related genes (TERT, IL2RA, and CCDC26) were screened and used to establish the prognostic model (Fig. 5C).

**Figure 6:**
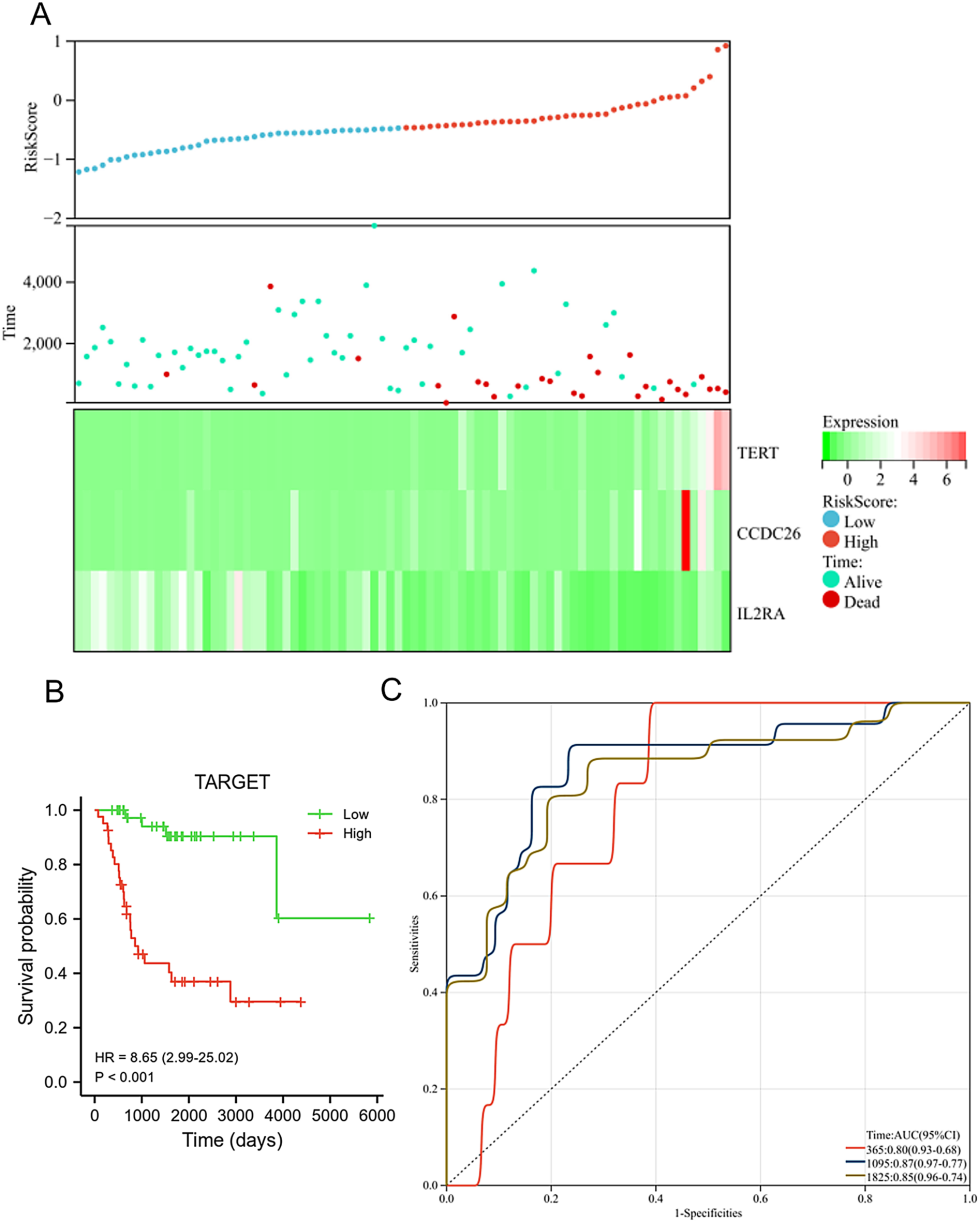
Assessment of prognostic model in the TARGET database. (**A**) The expression level of TERT, CCDC26 and IL2RA (below), survival status (middle), and the distribution of risk scores between low and high-risk groups (upper). (**B**) Survival analysis of showed the difference between low and high-risk groups. (**C**) Time-dependent ROC curve analyses of the prognostic model.

**Figure 7:**
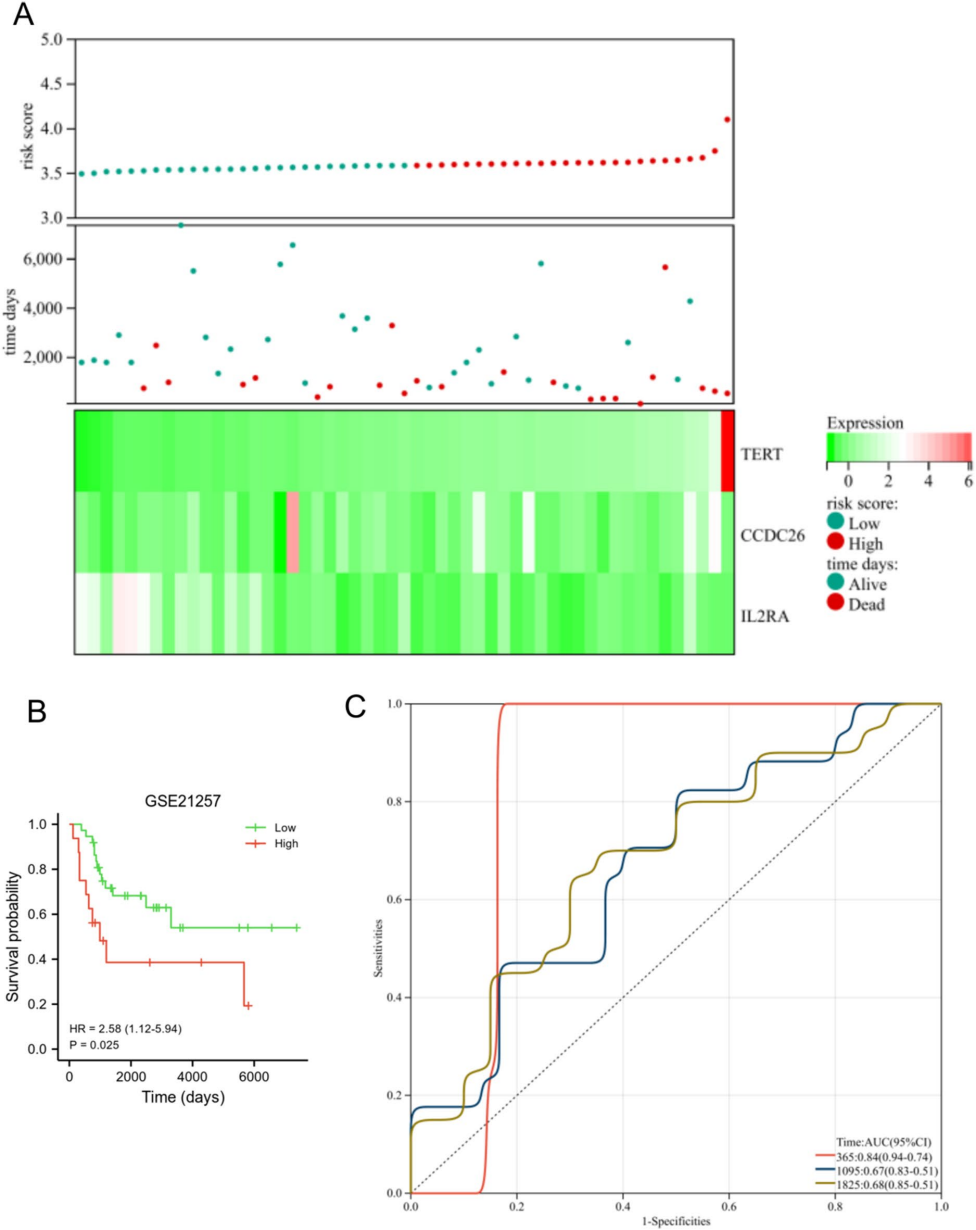
Validation of prognostic model in the GSE21257 dataset. (**A**) The expression level of TERT, CCDC26 and IL2RA (below), survival status (middle), and the distribution of risk scores between low and high-risk groups (upper). (**B**) Survival analysis of showed the difference between low and high-risk groups. (**C**) Time-dependent ROC curve analyses of the prognostic model.

In the TARGET dataset, the expression level of TERT and CCDC26 was down-regulated in the low risk group, while the expression of IL2RA was up-regulated in the low risk group. Moreover, high risk group had a lower proportion of alive (Fig. 6A). OS patients in the low risk group exhibited longer overall survival than those in the high risk group (Fig. 6B, *p* < 0.001). The AUC for this prognostic model was 0.8 at 1-year, 0.87 at 3-year, and 0.85 at 5-year (Fig. 6C), this result indicated that the prognostic model had good diagnostic performance for OS patients. We also used the GSE21257 dataset to verify the diagnosis and prognostic features of the risk model. As shown in Fig. 7A, the expression level of TERT and CCDC26 was down-regulated in the low-risk group, while the expression of IL2RA was up-regulated in the low-risk group. Moreover, high risk group had a lower proportion of alive. OS patients in the low-risk group exhibited longer overall survival than those in the high-risk group (*p* = 0.025) (Fig. 7B). The AUC for this prognostic model was 0.84 at 1-year, 0.67 at 3-year, and 0.68 at 5-year (Fig. 7C). These results were consistent with the results in the TARGET dataset.

**Figure 8:**
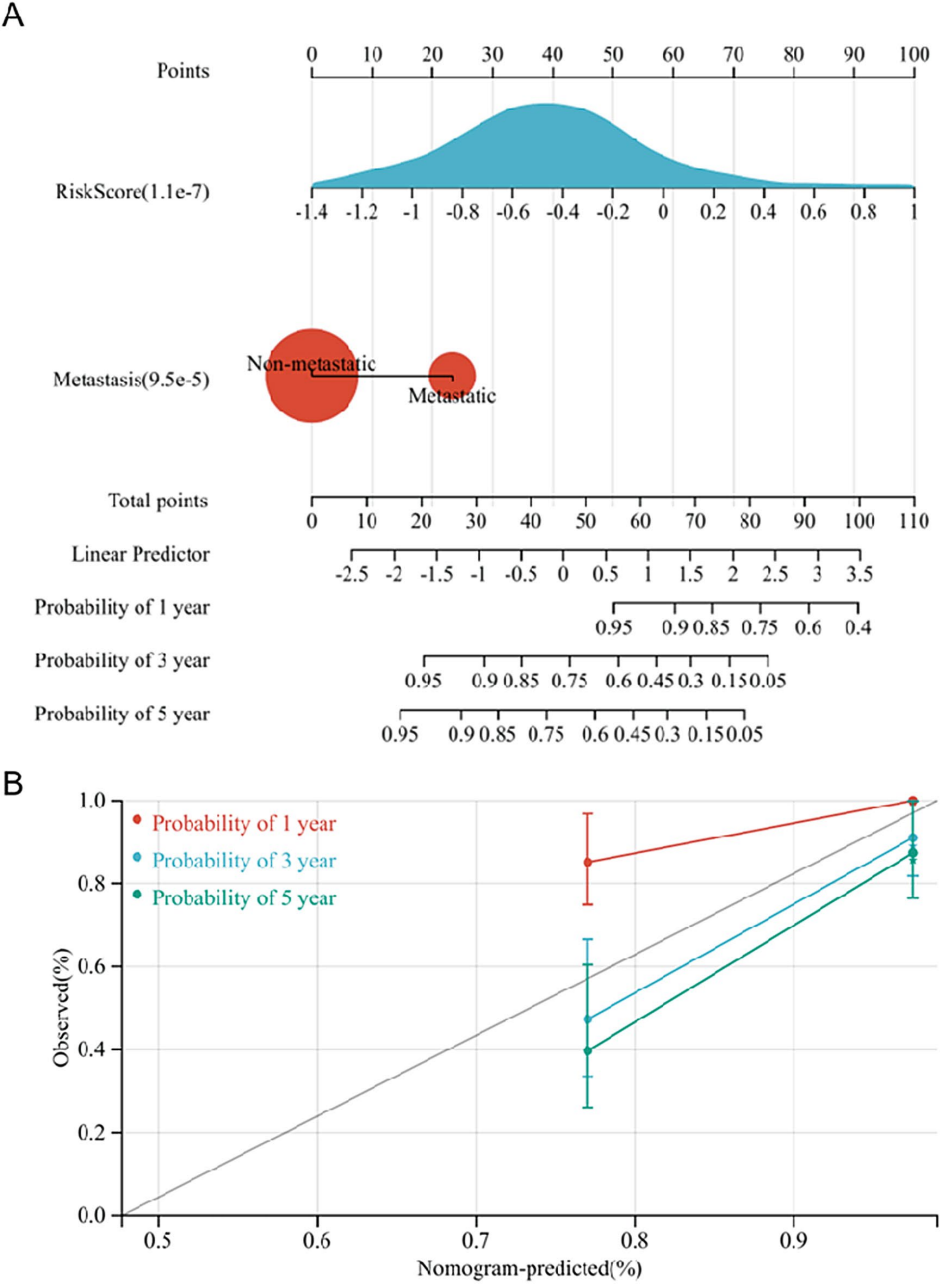
Establishment of the nomogram. (**A**) Metastasis and risk score were used to establish the nomogram. (**B**) Calibration curve of the nomogram.

In addition, a nomogram was established to further aid in predicting the prognosis of OS patients (Fig. 8A). The prediction results of the nomogram were highly consistent with the observation of OS patients based on the nomogram calibration curve (Fig. 8B).

### Interaction analysis of prognostic genes

**Figure 9:**
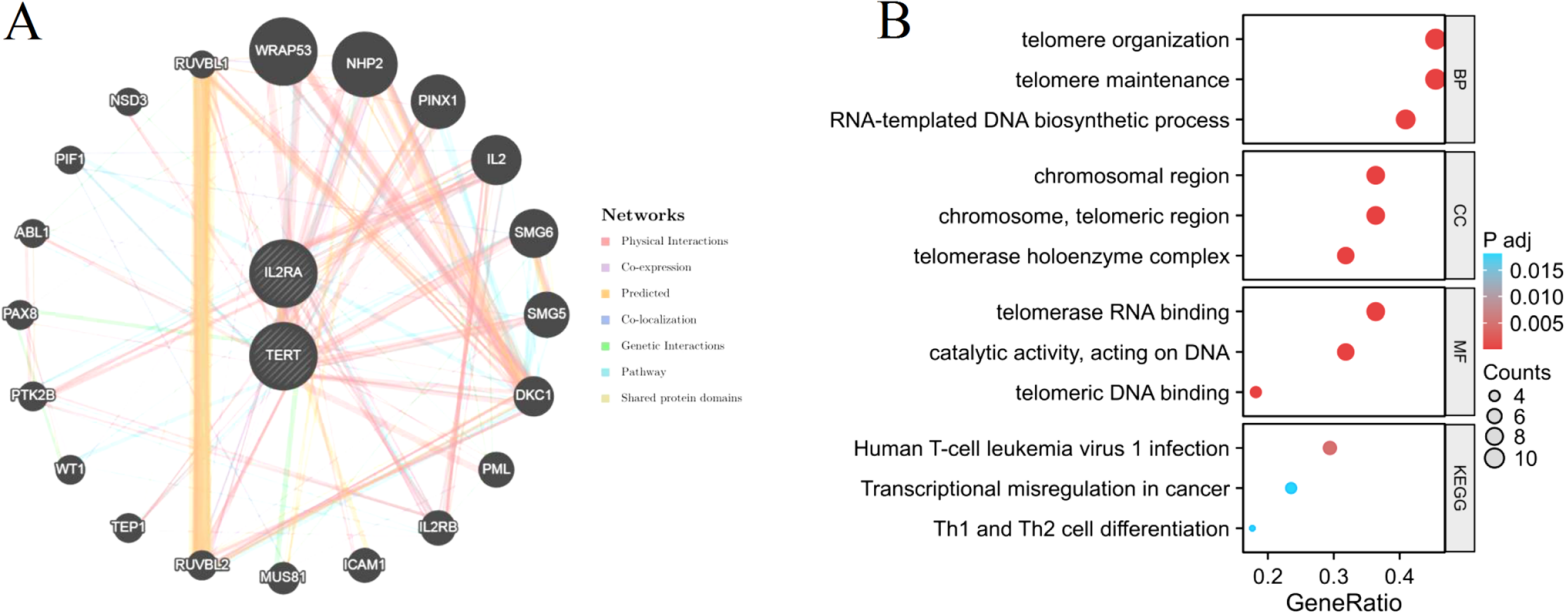
Interaction analysis of signature genes. (**A**) The signature genes' co-expression network. (**B**) Functional enrichment analysis of co-expressed genes through GO and KEGG.

By employing the GeneMANIA database, we successfully built a protein interaction network for the signature genes (TERT and IL2RA). Through this analysis, we discovered a total of 20 genes that engage in interactions with the signature genes (Fig. 9A). Functional enrichment analysis was conducted on these 22 genes. The outcomes obtained from the enrichment analysis demonstrated that these genes are predominantly linked to the telomere organization, telomere maintenance, human T cell leukemia virus 1 infection, Th1 and Th2 cell differentiation, etc. (Fig. 9B).

### Association between risk model-related genes and tumor microenvironment (TME)

**Figure 10:**
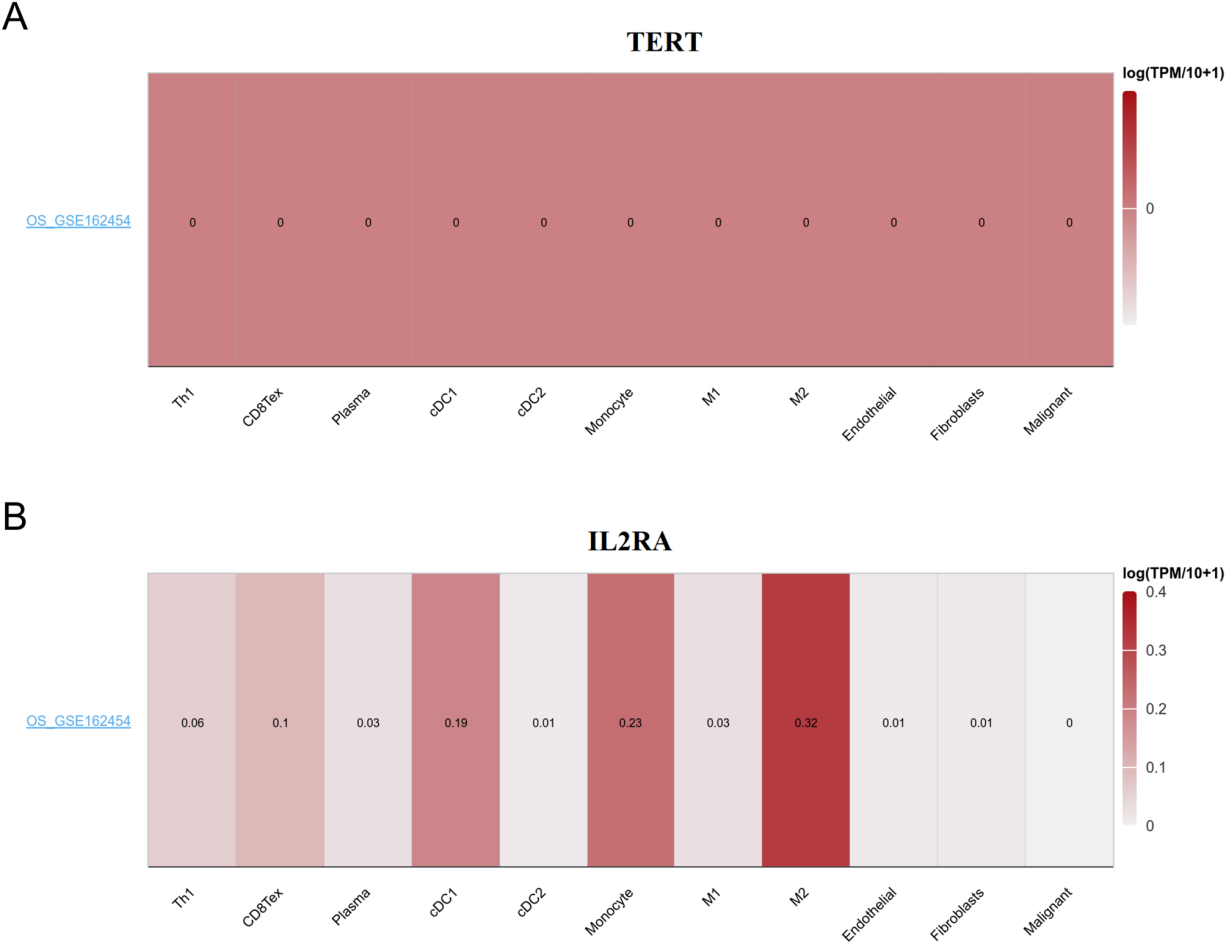
Genes associated with risk models were expressed in cells that are relevant to the tumor microenvironment. The expression levels of TERT (**A**) and IL2RA (**B**) in OS microenvironment-related cells were visualized using a heatmap in the dataset GSE162454.

We conducted an analysis on the expression levels of the TERT and IL2RA genes in tumor microenvironment-associated cells within OS, using the TISCH database. Our findings demonstrated that IL2RA displayed a higher level of infiltration in cDC1, monocyte, and M2 cells (Fig. 10).

### The prognostic model has the ability to distinguish metastatic OS patients

**Figure 11:**
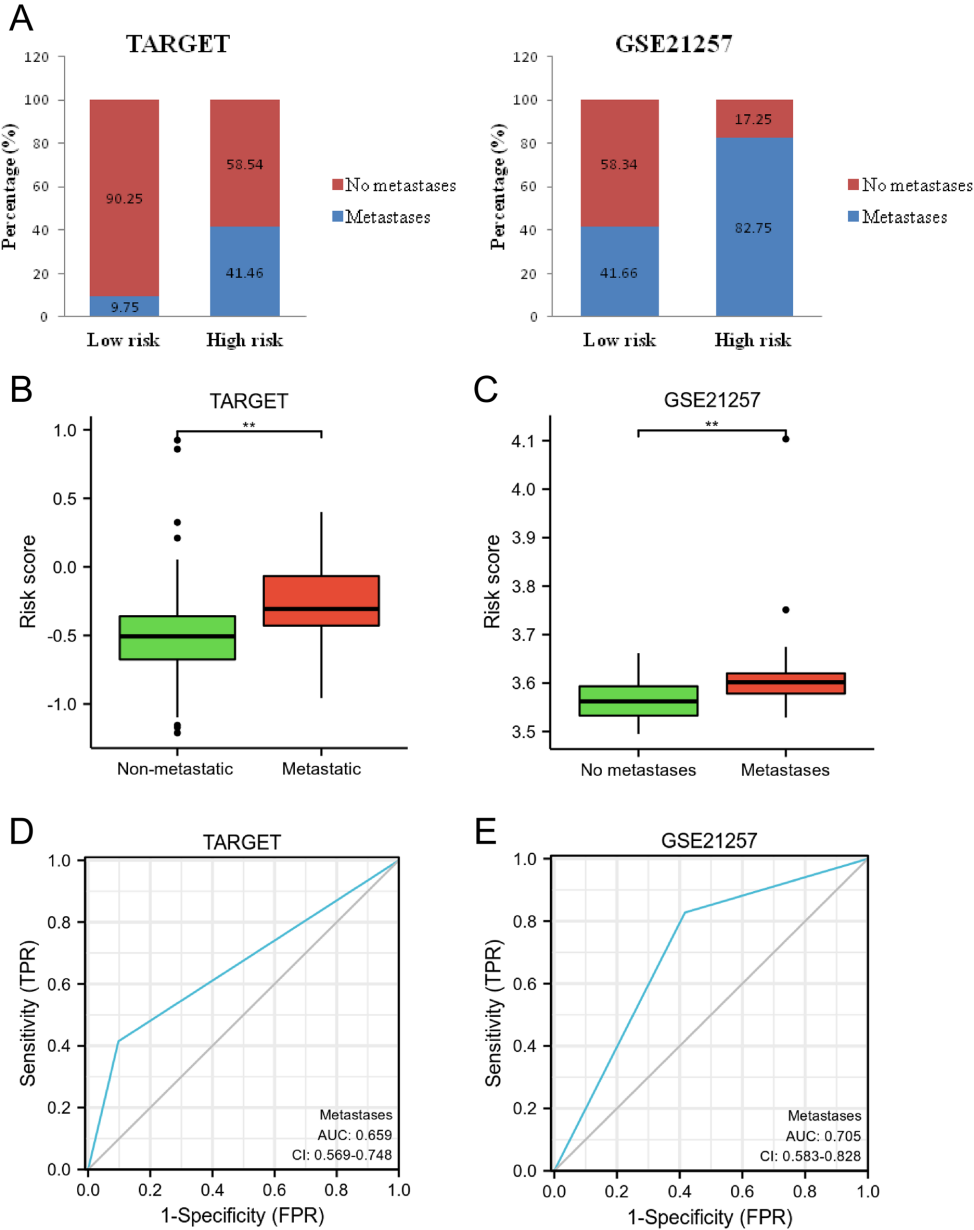
Assessment of the ability of prognostic model to predict metastasis in patients with OS. (**A**) The comparisons of metastasis and no metastasis cases in the low and high-risk groups. (**B**) The comparisons of risk score in the metastatic and non-metastatic groups. (**C**) ROC analysis for the diagnostic performance in the prediction of OS metastasis.

As shown Fig. 11A, compared to the high-risk group (TARGET, 58.54% and GSE21257, 17.25%), more no metastasis cases (TARGET, 90.25% and GSE21257, 58.34%) were observed in the low-risk group. Moreover, compared with the metastatic group, the risk score was lower in the non-metastatic group (*p* < 0.01, Fig. 11B,C). Furthermore, the results of ROC analysis showed that the diagnostic performance of the prognostic model for the prediction of metastasis were 0.659 and 0.705 in TARGET and GSE21257, respectively (Fig. 11D,E). These findings showed that the risk model could predict metastasis in OS patients.

## Discussion

OS is a highly malignant cancer, and 80% of OS patients still died from metastasis^5,23,24^. Therefore, its treatment still faces great challenges. Recent studies have demonstrated that several diagnostic, management, and prognostic analyzes associated with immune cell populations provided clinical guidance for improving treatment outcomes in patients with OS^25–27^. Furthermore, a bioinformatics-based research method, including sample collection, genomic analysis, and identification of regulatory networks, can promote a better deciphering and understanding of the underlying pathological mechanisms of action of the immune system in OS^28–30^. Thus, identifying genes related to immune cell infiltration is important for improving the treatment and diagnosis of OS patients.

In this study, we conducted a comprehensive analysis to investigate the potential impact of ML-related genes as prognostic indicators. We have successfully uncovered novel insights into the survival-related ML-related genes of OS. Throughout this research, we have made several groundbreaking discoveries. Firstly, we have successfully pinpointed 435 DEGs between ML-related subgroups, with a majority of them being involved in immune- and inflammation-related pathways. Secondly, we have successfully devised a ML-related signature and established a scoring system that exhibits a significant correlation with the overall survival of OS patients. This unique signature has proven to be highly effective in accurately stratifying patients into low- and high-risk groups, while also providing precise predictions for the overall survival of OS patients with sensitivity and specificity. Our findings in the field of OS are consistent with the existing literature^31^. Previous study has reported similar patterns of gene expression alterations and identified signature genes classifier in OS patients. Our study further supported the notion that the signature genes play crucial roles in OS development and progression. Furthermore, our enrichment analyses results provided additional insights into the potential mechanisms of these signature genes, highlighting their potential as therapeutic targets in OS treatment.

In our research, the immunocyte infiltration level in OS patients was assessed through the MCP-counter. Our results indicated that a low level of monocytic lineage (ML) was related to a bad prognosis in OS patients. Monocytes are important immune cells and important regulators of cancer initiation and progression^32^. The monocyte-directed adjuvant therapies had the potential value in the treatment of cancer^33,34^. A recent study showed that increased peripheral blood monocytes counts were associated a poorer prognosis in pancreatic cancer patients^35^. Immune cell infiltration involves in OS metastasis, and patrolling monocytes suppressed OS metastasis to lungs of mice^36^. The results mentioned above suggested that the activation of monocytes might play a role in improving the overall survival rate of OS patients. Nonetheless, additional experiments are needed to validate the accuracy of this conclusion.

Another important finding is that we established a prognostic diagnostic model for OS patients. We identified 435 ML-related genes, and three genes (TERT, CCDC26, and IL2RA) were used to construct the prognostic model. Telomerase (TERT) was a catalytic subunit of telomerase, which involves in tumorigenesis^37^. TERT gene exerted carcinogenic effect, targeting TERT was an effective therapy in the treatment of non-small cell lung cancer^38^. TERT could potentially function as a valuable genomic indicator for detecting and forecasting various types of cancer, while also holding promise as a potential target for therapeutic interventions in the case of OS^39^. In addition, TERT was a potential prognostic biomarker in diagnosis and prognosis of cancer, including breast cancer, thyroid cancer, and bladder cancer^40–42^. Moreover, the suppression of TERT expression decreased osteosarcoma cell metastasis, motility, and proliferation^39^. At present, the role of Coiled-coil domain containing (CCDC26) in cancer prognosis remains unexplored. CCDC26 knockdown could cause imatinib resistance in gastrointestinal stromal tumor cells through decreasing c-KIT expression^43^. CCDC26 rs4295627 polymorphism was a risk marker for glioma patients^44,45^. Interleukin 2 receptor subunit alpha (IL2RA) was increased after stimulation in immune cells, such as regulatory T cells^46^. Recent studies revealed that IL2RA was closely related to the development and progression of tumorigenesis and the prognosis of cancer patients. IL2RA suppressed differentiation and contributed to stem cell-related properties, implying that it was a potential target in acute myeloid leukemia^47^. In addition, elevated mRNA expression of IL2RA was an adverse prognostic biomarker in acute myeloid leukemia^48^. Higher expression of IL2RA was related to the poorer prognosis and higher immune infiltration level in pancreatic ductal adenocarcinoma^49,50^. IL2RA is a molecule that is relatively recent in its discovery, with only limited studies on its involvement in OS currently available. However, it has been found to play a significant part in the development of tumors. Additional investigations are warranted to thoroughly investigate the extent of its impact on OS. In our study, the prognostic model containing TERT, CCDC26, and IL2RA genes, and it showed good prognostic performance, as well as for the prediction of metastasis in OS patients. Therefore, this prognostic risk model is helpful for the diagnosis and treatment of OS patients.

There were several limitations in this study. The study primarily focused on data mining and data analysis, the results was not validated by external experiments. Further research is needed to validate our findings.

## Conclusion

In general, two ML-associated molecular subtypes and a ML-related signature (TERT, CCDC26, and IL2RA) were identified for the establishment of the prognostic model. The low infiltration level of ML was related to a bad prognosis and inactivated immune status in OS patients. Moreover, the ML-related prognostic model could predict prognosis and metastasis in OS patients.

### Supplementary Information

Supplementary Information.

## Data Availability

Any data not included in the article or supplementary data will be made available upon request to the corresponding author. The data used in our study are available from the GSE21257 dataset (https://www.ncbi.nlm.nih.gov/geo/geo2r/?acc=GSE21257), and TARGET database (https://ocg.cancer.gov/programs/target).
